# Interaction Between Vitamin D Status and Aerobic Physical Activity in Relation to Cardiometabolic Risk Clustering Among Rural Older Adults

**DOI:** 10.3390/healthcare14040543

**Published:** 2026-02-22

**Authors:** Kyeongmin Jang, Hye Won Yun, Sung Hwan Kim

**Affiliations:** 1 Department of Nursing, College of Health Sciences, Daejin University, 1007 Hoguk-ro, Pocheon-si 11159, Gyeonggi-do, Republic of Korea; kyeongminjang@gmail.com; 2 Department of Nursing, Catholic Sangji University, 45 Sangji-gil, Andong-si 36686, Gyeongsangbuk-do, Republic of Korea; dntntn20@naver.com

**Keywords:** vitamin D, 25-hydroxyvitamin D, aerobic physical activity, cardiometabolic risk clustering, effect modification, interaction, rural older adults

## Abstract

**Highlights:**

**What are the main findings?**

- Among rural Korean adults aged ≥65 years (KNHANES 2023, *n* = 441), serum 25(OH)D significantly modified the association between meeting WHO aerobic physical activity guidelines and CMRC (interaction OR = 0.91, 95% CI: 0.85–0.97).
- Physical activity was linked to markedly lower odds of CMRC only at higher vitamin D levels (84th percentile 36.98 ng/mL: OR = 0.25, 95% CI 0.10–0.62), but not at low vitamin D levels (16th percentile 17.08 ng/mL: OR = 1.64, 95% CI 0.78–3.47).

**What are the implications of the main findings?**

- Cardiometabolic prevention in rural older adults may be more effective when aerobic activity promotion is paired with strategies to address vitamin D insufficiency, rather than relying on activity counseling alone.
- Incorporating vitamin D status into community screening and risk stratification could help target integrated, resource-efficient interventions for high-risk rural aging populations.

**Abstract:**

Background/Objectives: This study investigated whether serum 25-hydroxyvitamin D (25(OH)D) modifies the association between meeting WHO aerobic physical activity guidelines and cardiometabolic risk clustering among rural older adults in South Korea. Methods: This cross-sectional study analyzed 2023 Korea National Health and Nutrition Examination Survey (KNHANES) data for rural-dwelling adults aged ≥65 years with complete data (*n* = 441). Cardiometabolic risk clustering (CMRC) was defined as the presence of ≥3 of five risk factors (abdominal obesity, elevated blood pressure, low HDL-cholesterol, elevated triglycerides, and hyperglycemia). Exposures were continuous serum 25-hydroxyvitamin D (25(OH)D) and adherence to the WHO aerobic physical activity guidelines (yes/no). Multivariable logistic regression models tested the 25(OH)D × physical activity interaction, adjusting for sex, age (≥75 vs. <75 years), education, household income, smoking status, alcohol use, and obesity (BMI ≥ 25 kg/m^2^). Conditional effects of physical activity were estimated at the 16th, 50th, and 84th percentiles of 25(OH)D. Results: A significant interaction between 25(OH)D and physical activity was observed (OR = 0.91, 95% CI: 0.85–0.97; *p* = 0.005). Physical activity was not associated with CMRC at low 25(OH)D (16th percentile, 17.08 ng/mL; OR = 1.64, 95% CI: 0.78–3.47), but it was associated with lower odds of CMRC at high 25(OH)D (84th percentile, 36.98 ng/mL; OR = 0.25, 95% CI: 0.10–0.62). Conclusions: Vitamin D status modified the association between aerobic physical activity and cardiometabolic risk clustering among rural older adults. Integrated prevention strategies addressing both physical activity and vitamin D insufficiency may be valuable in rural aging populations.

## 1. Introduction

Cardiometabolic risk tends to accumulate with age, and the co-occurrence of multiple abnormalities—such as central adiposity, elevated blood pressure, dyslipidemia, and impaired glucose regulation—substantially increases the likelihood of future cardiovascular disease and type 2 diabetes compared with isolated risk factors [1]. In population studies, defining “risk clustering” as the presence of three or more cardiometabolic risk factors has been widely used to identify individuals with a high-risk profile and to describe how these risks aggregate within individuals [2].

Older adults living in rural areas may be particularly vulnerable to cardiometabolic risk clustering because they often experience a higher burden of chronic conditions and face structural barriers to health-promoting behaviors and preventive care [3,4]. In South Korea, national surveillance data show that insufficient aerobic physical activity remains common and has shown an unfavorable trend over time, underscoring the need to clarify modifiable determinants of cardiometabolic risk among older adults [5]. Physical activity is a cornerstone behavior for cardiometabolic prevention, and the WHO 2020 guidelines recommend at least 150–300 min/week of moderate-intensity or 75–150 min/week of vigorous-intensity aerobic activity (or an equivalent combination) for adults, including older adults [6].

Alongside lifestyle behaviors, vitamin D status has been repeatedly examined as a potentially relevant factor for cardiometabolic health in observational epidemiology. Recent epidemiologic studies have reported associations between circulating 25(OH)D and cardiometabolic risk profiles, although residual confounding remains a key concern [7]. Physical activity and vitamin D are also behaviorally linked, because higher physical activity—particularly when performed outdoors—may reflect greater sunlight exposure, which is a key determinant of circulating 25(OH)D levels [8]. From a biological perspective, vitamin D has been implicated in molecular pathways related to insulin sensitivity, lipid metabolism, and inflammatory processes, providing a plausible rationale for considering vitamin D status in cardiometabolic risk profiling [9].

A recent KNHANES-based study in middle-aged adults (40–64 years) examined independent associations of vitamin D and C-reactive protein with cardiometabolic risk clustering (CMRC) [10]. However, despite extensive research on vitamin D and physical activity as individual correlates of cardiometabolic outcomes, evidence remains limited on whether vitamin D modifies the association between adherence to WHO aerobic physical activity guidelines and CMRC in older adults, particularly among rural-dwelling older adults. Because physical activity and adiposity are tightly linked to both vitamin D status and cardiometabolic risk, clarifying this potential effect modification is methodologically and clinically relevant [7]. In practical terms, this approach may help identify subgroups for whom meeting aerobic activity recommendations is more strongly linked to lower CMRC, informing more targeted prevention strategies.

Given rapid population aging in Korea, identifying modifiable correlates of cardiometabolic risk clustering in rural older adults has direct public health relevance for community-based prevention and resource allocation. Therefore, this study investigated whether serum 25(OH)D modifies the association between meeting WHO aerobic physical activity guidelines and cardiometabolic risk clustering among rural Korean adults aged ≥65 years using the 2023 KNHANES (released December 2024). Specifically, we (1) described participant characteristics and differences by vitamin D status, (2) examined factors associated with cardiometabolic risk burden using a CMRC count score, and (3) tested whether serum vitamin D modified the association between meeting aerobic physical activity guidelines and the odds of CMRC (≥3 risk factors).

## 2. Materials and Methods

### 2.1. Study Design

This cross-sectional study used data from the 2023 Korea National Health and Nutrition Examination Survey (KNHANES), which was conducted in 2023 and publicly released in December 2024. The KNHANES sampling design was considered in the analyses to support population-representative inference [11].

### 2.2. Participants and Data Source

The 2023 KNHANES dataset included 6929 participants across all ages, of whom 1836 were aged ≥65 years. We restricted the analytic sample to rural-dwelling older adults (≥65 years) (*n* = 527), classified as rural based on the KNHANES administrative district classification. Specifically, participants residing in eup or myeon areas were classified as rural, whereas those residing in dong areas were classified as urban. After excluding 86 participants with missing data on the outcome, exposures, or covariates required for the multivariable models, the final analytic sample comprised 441 rural-dwelling older adults. KNHANES data were collected by trained staff using standardized health interviews, examinations, and laboratory testing protocols.

### 2.3. Variables and Operational Definitions

The primary outcome was CMRC, operationalized in two complementary ways. First, a binary CMRC variable was defined as the presence of three or more cardiometabolic risk factors and was used as the main outcome for interaction modeling. Second, a count CMRC score was calculated as the sum of risk factors (range 0–5) and analyzed as a secondary continuous outcome.

The five cardiometabolic risk factors were defined as follows: abdominal obesity (waist circumference ≥90 cm for men and ≥85 cm for women, based on the Korean obesity guideline definition [12]), elevated blood pressure (systolic blood pressure ≥ 130 mmHg, diastolic blood pressure ≥ 85 mmHg, or current use of antihypertensive medication), low HDL-cholesterol (<40 mg/dL in men and <50 mg/dL in women), elevated triglycerides (≥150 mg/dL or current use of lipid-lowering medication), and hyperglycemia (fasting plasma glucose ≥ 100 mg/dL or current use of antidiabetic medication).

The main exposures were serum vitamin D and physical activity. Serum vitamin D was assessed as total 25-hydroxyvitamin D [25(OH)D] concentration (ng/mL). For primary analyses, 25(OH)D was treated as a continuous variable and mean-centered for interaction models. For descriptive comparisons and selected regression models, vitamin D status was additionally categorized as <20 ng/mL vs. ≥20 ng/mL, a threshold commonly applied in epidemiologic studies examining cardiometabolic risk [13].

Physical activity was assessed using the KNHANES physical activity questionnaire (the Korean version of the Global Physical Activity Questionnaire). Adherence to the WHO aerobic physical activity recommendation was defined as ≥150 min/week of moderate-intensity activity, ≥75 min/week of vigorous-intensity activity, or an equivalent combination (moderate + 2× vigorous), and was coded as a binary variable (meeting vs. not meeting the recommendation) [6]. Because GPAQ-derived estimates may primarily capture activity accumulated in bouts of at least 10 min, our operational definition may not fully reflect the WHO 2020 guidance allowing accumulation in bouts of any duration.

Covariates were selected a priori as potential confounders of the relationships among vitamin D, physical activity, and CMRC. These included age (≥75 vs. <75 years), sex, education (middle school or higher vs. below), household income (middle–high vs. low), current smoking (yes vs. no), current alcohol use (yes vs. no), and general obesity (BMI ≥25 kg/m^2^ vs. <25 kg/m^2^). Laboratory lipid and glycemic measures were used to construct the CMRC outcome variables and were not additionally entered as covariates to avoid adjusting for variables that directly define the outcome, which can bias effect estimates or reduce interpretability in observational models [14].

### 2.4. Statistical Analysis

Continuous variables are presented as mean ± standard deviation, and categorical variables as *n* (%). Differences by vitamin D status (<20 vs. ≥20 ng/mL) were examined using independent-samples t tests and Pearson’s chi-square tests. Multivariable linear regression was performed for the CMRC count score (0–5), and multivariable logistic regression for binary CMRC (≥3 vs. <3). The logistic model included a vitamin D × physical activity interaction; serum 25(OH)D was mean-centered. Conditional effects of physical activity were estimated at the 16th percentile, median, and 84th percentile of vitamin D and are reported as odds ratios with 95% confidence intervals. All models were adjusted for sex, age (≥75 vs. <75), education, income, smoking, alcohol use, and obesity (BMI ≥25 kg/m^2^). Statistical significance was set at *p* < 0.05 (two-sided).

### 2.5. Ethical Considerations

KNHANES was conducted by the KDCA in accordance with the Declaration of Helsinki and was approved by the KDCA Institutional Review Board (IRB No. 2022-11-16-R-A; approval date: 16 November 2022). Written informed consent was obtained from all participants at the time of the original survey. This study involved secondary analysis of publicly available KNHANES data collected in 2023 and released in December 2024. The dataset is fully de-identified and contains no personally identifiable information. In accordance with applicable regulations and institutional policies for the use of public, de-identified data, additional IRB review was not required for this analysis.

## 3. Results

### 3.1. Characteristics of the Study Participants

A total of 441 rural-dwelling older adults aged ≥65 years were included in the analysis (Table 1). The mean age was 72.57 ± 5.23 years, and 201 participants (45.6%) were men. The mean waist circumference was 86.55 ± 9.35 cm, and the mean body mass index (BMI) was 24.16 ± 3.28 kg/m^2^. Abdominal obesity was present in 224 participants (50.8%).

The mean serum 25-hydroxyvitamin D concentration was 27.74 ± 10.84 ng/mL. Mean fasting plasma glucose was 105.24 ± 24.14 mg/dL, and mean hemoglobin A1c was 5.90 ± 0.87%. The mean triglyceride level was 123.64 ± 67.33 mg/dL, total cholesterol was 174.61 ± 40.91 mg/dL, and HDL cholesterol was 53.02 ± 13.54 mg/dL (Table 1).

With respect to comorbidities, hypertension was reported in 253 participants (57.4%), diabetes mellitus in 92 participants (20.9%), and dyslipidemia in 183 participants (41.5%). Chronic kidney disease and asthma were reported in 17 (3.9%) and 23 participants (5.2%), respectively (Table 1).

### 3.2. Differences in Characteristics by Vitamin D Status

Participants were classified according to serum vitamin D status as <20 ng/mL (*n* = 110, 24.9%) or ≥20 ng/mL (*n* = 331, 75.1%) (Table 2). Participants with vitamin D < 20 ng/mL were older than those with levels ≥20 ng/mL (73.43 ± 5.50 vs. 72.28 ± 5.12 years, *p* = 0.046). The proportion of men did not differ significantly between the two groups (40.9% vs. 43.5%, *p* = 0.641).

No significant differences were observed between the two groups in education level (*p* = 0.182), household income (*p* = 0.159), living alone (*p* = 0.175), current smoking status (*p* = 0.497), alcohol use (*p* = 0.310), or physical inactivity (*p* = 0.374) (Table 2). Mean BMI (24.09 ± 3.43 vs. 24.63 ± 3.15 kg/m^2^, *p* = 0.148) and waist circumference (87.92 ± 10.16 vs. 86.09 ± 9.04 cm, *p* = 0.076) were also similar between groups.

Among laboratory parameters, participants with vitamin D < 20 ng/mL had higher triglyceride levels than those with ≥20 ng/mL (140.67 ± 81.71 vs. 117.98 ± 60.92 mg/dL, *p* = 0.002). No significant group differences were observed in total cholesterol (*p* = 0.385), HDL cholesterol (*p* = 0.381), fasting glucose (*p* = 0.979), or hemoglobin A1c (*p* = 0.952) (Table 2).

The prevalence of cardiometabolic risk clustering (CMRC ≥ 3) was higher in the vitamin D–deficient group than in the non-deficient group (47.3% vs. 34.1%, *p* = 0.014). The prevalence of hypertension, diabetes mellitus, hyperlipidemia, chronic kidney disease, and asthma did not differ significantly by vitamin D status (all *p* > 0.05) (Table 2).

### 3.3. Factors Associated with CMRC Count Score

Multivariable linear regression analysis was performed with the CMRC count score (0–5) as the dependent variable (Table 3). After adjustment for covariates, vitamin D ≥20 ng/mL was inversely associated with CMRC count (B = −0.121, SE = 0.049, β = −0.108, *p* = 0.014). Obesity (BMI ≥ 25 kg/m^2^) was positively associated with CMRC count (B = 0.435, SE = 0.043, β = 0.437, *p* < 0.001). Sex, age ≥75 years, education level, household income, physical activity, current smoking, and alcohol use were not significantly associated with CMRC count (all *p* > 0.05) (Table 3). The model explained 21.7% of the variance in CMRC count (adjusted R^2^ = 0.201).

### 3.4. Interaction Between Vitamin D and Physical Activity on CMRC

Table 4 presents the results of the multivariable logistic regression analysis examining the association between physical activity, serum vitamin D, and binary CMRC (≥3 risk factors). In the interaction model, the vitamin D × physical activity interaction term was statistically significant (OR = 0.91, 95% CI: 0.85–0.97, *p* = 0.005) (Table 4, A).

Conditional effects of physical activity at representative vitamin D levels are shown in Table 4, B. At a low vitamin D level (16th percentile, 17.08 ng/mL), physical activity was not significantly associated with CMRC (OR = 1.64, 95% CI: 0.78–3.47, *p* = 0.192). At the median vitamin D level (27.48 ng/mL), the association approached statistical significance (OR = 0.61, 95% CI: 0.36–1.03, *p* = 0.066). At a high vitamin D level (84th percentile, 36.98 ng/mL), physical activity was significantly associated with lower odds of CMRC (OR = 0.25, 95% CI: 0.10–0.62, *p* = 0.003).

## 4. Discussion

In this cross-sectional study of rural-dwelling older adults in Korea, we observed a significant interaction between serum vitamin D status and physical activity in relation to cardiometabolic risk clustering (CMRC). Vitamin D sufficiency (≥20 ng/mL) was independently associated with a lower cardiometabolic risk burden, as reflected by a reduced CMRC count score. Importantly, meeting aerobic physical activity guidelines was associated with substantially lower odds of CMRC only at higher vitamin D levels, suggesting that vitamin D may modify the relationship between physical activity and clustered cardiometabolic risk.

### 4.1. Vitamin D and Physical Activity in Cardiometabolic Risk

Numerous observational studies have reported inverse associations between circulating 25(OH)D levels and cardiometabolic risk profiles, including metabolic syndrome. Recent meta-analyses suggest that lower serum vitamin D concentrations are associated with a higher prevalence of cardiometabolic abnormalities, though effect sizes are modest and heterogeneity remains substantial [15,16].

Similarly, physical activity has been consistently linked to favorable cardiometabolic profiles in older adults, including reduced abdominal obesity, improved lipid profiles, and better glucose regulation [17]. Evidence from Korean population-based studies further indicates that adherence to aerobic activity guidelines is associated with lower cardiometabolic risk; however, adherence remains low among older adults and has shown an unfavorable trend over time [18,19]. Despite these well-established individual associations, few studies have examined whether vitamin D status modifies the relationship between physical activity and cardiometabolic risk clustering. Our findings address this gap by supporting effect modification, rather than a purely additive relationship, between vitamin D status and physical activity.

### 4.2. Interpretation and Potential Mechanisms

The observed interaction between vitamin D and physical activity may be understood through both behavioral and biological mechanisms. Behaviorally, aerobic activity—particularly when performed outdoors—may reflect increased sunlight exposure, a key determinant of endogenous vitamin D synthesis; population-based evidence also supports links between circulating 25(OH)D and broader health outcomes [20]. In addition, physical activity and adiposity are jointly related to vitamin D status, implying that individuals who are both active and vitamin D sufficient may reflect an overall healthier cardiometabolic profile [21]. In addition, the descriptive comparisons showed that participants with vitamin D < 20 ng/mL were slightly older and had higher triglyceride levels than those with ≥20 ng/mL. Older age may be linked to reduced time spent outdoors and lower sunlight exposure, which can contribute to lower 25(OH)D concentrations [8]. Higher triglycerides in the vitamin D-deficient group may reflect a less favorable metabolic profile that tends to cluster with vitamin D insufficiency in observational data [15]. Importantly, age and other sociodemographic and behavioral factors were adjusted for in the multivariable models; nevertheless, residual confounding cannot be fully excluded [14].

Biologically, vitamin D has been implicated in mechanisms regulating insulin sensitivity, lipid metabolism, and inflammation. Recent mechanistic and narrative reviews support potential roles of vitamin D in pathways related to insulin resistance and metabolic dysfunction [22,23]. While causality cannot be inferred from the present cross-sectional design, this interpretation is consistent with evidence emphasizing that adiposity and related factors are tightly interwoven with vitamin D status and cardiometabolic risk, raising the possibility of residual confounding in observational research [24,25].

To aid interpretation of the interaction effect size, it is important to consider the scaling of serum 25(OH)D in the model. Because 25(OH)D was treated as a continuous variable, the interaction odds ratio (OR = 0.91) represents the change in the physical activity–CMRC association per 1 ng/mL increase in 25(OH)D and therefore appears numerically close to 1. However, when evaluated across clinically meaningful differences in vitamin D levels, the conditional estimates indicated substantial variation in the association, with markedly lower odds of CMRC among those meeting aerobic activity guidelines at higher vitamin D percentiles but not at lower levels. These contrasts support the interpretation that vitamin D status may meaningfully modify the association between aerobic physical activity and clustered cardiometabolic risk in this population.

### 4.3. Implications for Public Health Nursing

From a public health nursing perspective, these findings have practical relevance for rural older adults, who often face a higher cardiometabolic burden alongside structural barriers to preventive services. The observed effect modification suggests that “one-size-fits-all” physical activity promotion may yield uneven benefits; rather, identifying subgroups in whom meeting aerobic activity guidelines is most strongly associated with lower cardiometabolic risk can support more targeted, context-sensitive strategies. In rural communities, public health nurses working through local public health centers and community health posts are well-positioned to implement integrated prevention efforts that specify who should be prioritized, where services can be delivered, and what components are needed. Specifically, routine outreach encounters (e.g., chronic disease follow-up, home visits, and community screening events) can incorporate (1) brief aerobic physical activity counseling and referral tailored to functional status and safety [6], (2) screening to identify individuals at higher risk of vitamin D insufficiency—such as those with low 25(OH)D levels, limited outdoor time, or winter-season constraints—and (3) linkage to nutrition education and appropriate supplementation pathways, alongside guidance on safe sunlight exposure when feasible.

Importantly, our interaction results suggest that physical activity alone may be less strongly associated with lower CMRC when vitamin D status is low, supporting a dual-component approach in which aerobic activity promotion is paired with strategies to address vitamin D insufficiency. Such combined programming may be particularly relevant in rural settings where seasonal variation in sunlight exposure and limited access to nutrition services can exacerbate vitamin D inadequacy [5]. At a systems level, integrating vitamin D status into risk stratification within rural public health nursing programs could help prioritize individuals for more intensive counseling, follow-up, and referrals, thereby improving the efficiency of community-based cardiometabolic prevention in the context of rapid population aging.

### 4.4. Strengths and Limitations

This study has several strengths, including the use of a nationally representative health survey, standardized measurement of cardiometabolic risk factors, and the examination of cardiometabolic risk clustering using both binary and count-based outcomes. Focusing on rural-dwelling older adults addresses an important but understudied population in cardiometabolic research. Several limitations should also be acknowledged. First, the cross-sectional design precludes causal inference regarding the observed associations and interaction effects. Accordingly, reverse causality is plausible: older adults with a higher cardiometabolic risk burden may be less able or less likely to engage in aerobic physical activity due to functional limitations, symptoms, or clinical advice. In addition, cardiometabolic dysregulation may influence vitamin D metabolism and circulating 25(OH)D levels, which could partly affect the observed associations and interaction. Second, serum 25(OH)D was measured at a single time point and may not reflect long-term vitamin D status. Seasonal variation and short-term changes in sunlight exposure or related behaviors may have introduced measurement error, which could have attenuated the observed associations. Third, physical activity was assessed using the self-reported Korean version of the Global Physical Activity Questionnaire, and is therefore subject to recall and social desirability bias. Moreover, although the WHO 2020 guidelines allow aerobic activity to be accumulated in bouts of any duration, GPAQ-derived estimates may primarily capture activity accumulated in bouts of at least 10 min; thus, adherence to the WHO recommendation may have been underestimated. Such non-differential misclassification would likely attenuate the observed associations. Finally, although we adjusted for key sociodemographic and behavioral covariates, residual confounding cannot be excluded. Unmeasured factors such as dietary patterns, sunlight exposure, vitamin D supplementation, medication use (e.g., lipid-lowering agents), and renal function may influence both vitamin D status and cardiometabolic risk.

## 5. Conclusions

This study indicates that serum vitamin D status may modify the association between meeting aerobic physical activity guidelines and cardiometabolic risk clustering among rural-dwelling older adults in South Korea. Specifically, meeting aerobic physical activity guidelines was associated with lower odds of cardiometabolic risk clustering primarily among individuals with higher serum 25(OH)D levels.

These findings support the potential utility of integrated prevention strategies that address both physical activity and vitamin D insufficiency in rural aging populations. Because of the cross-sectional design, the temporal direction of these associations cannot be determined. Prospective longitudinal studies and intervention trials that jointly address aerobic physical activity and vitamin D status are needed to confirm causality and clarify underlying mechanisms.

## Figures and Tables

**Table 1:** Characteristics of the study participants (*n* = 441).

Variable	Value (*n*, %) or M ± SD
Sociodemographic Characteristics	
Age (years)	72.57 ± 5.232
Male sex	201 (45.6%)
Waist circumference (cm)	86.55 ± 9.353
Body mass index (kg/m^2^)	24.16 ± 3.280
Abdominal obesity (yes)	224 (50.8%)
Clinical and Laboratory Characteristics	
Vitamin D (ng/mL)	27.74 ± 10.841
Fasting glucose (mg/dL)	105.24 ± 24.143
Hemoglobin A1c (%)	5.90 ± 0.865
Triglycerides (mg/dL)	123.64 ± 67.331
Total cholesterol (mg/dL)	174.61 ± 40.914
HDL cholesterol (mg/dL)	53.02 ± 13.542
Comorbidities	
Hypertension, yes	253 (57.4%)
Diabetes mellitus, yes	92 (20.9%)
Dyslipidemia, yes	183 (41.5%)
Chronic kidney disease, yes	17 (3.9%)
Asthma, yes	23 (5.2%)

Note. Values are presented as *n* (%) for categorical variables and mean ± standard deviation (SD) for continuous variables. HDL = high-density lipoprotein.

**Table 2:** Differences in sociodemographic characteristics, lifestyle factors, and CMRC according to vitamin D status (*n* = 441).

**Variable**	**<20 ng/mL** **(*n* = 110)**	**≥20 ng/mL** **(*n* = 331)**	**t or χ^2^**	***p***
Sociodemographic characteristics				
Sex (Male)	45 (40.9%)	144 (43.5%)	0.221	0.641
Age (years)	73.43 ± 5.502	72.28 ± 5.120	1.997	0.046
Education (≤Middle school)	88 (80.0%)	244 (73.7%)	1.782	0.182
Income (1st quintile)	35 (31.8%)	81 (24.5%)	1.991	0.159
Living alone	35 (31.8%)	82 (24.8%)	1.843	0.175
Lifestyle factors				
Current smoker	9 (8.2%)	21 (6.3%)	0.461	0.497
Alcohol use (≥once/month)	24 (21.8%)	89 (26.9%)	1.032	0.310
Physical inactivity	69 (62.7%)	191 (57.7%)	0.797	0.374
Anthropometric and blood parameters				
BMI (kg/m^2^)	24.09 ± 3.433	24.63 ± 3.151	1.378	0.148
Waist circumference (cm)	87.92 ± 10.161	86.09 ± 9.040	1.781	0.076
Total cholesterol (mg/dL)	174.52 ± 40.968	174.63 ± 40.956	−0.026	0.385
Triglycerides (mg/dL)	140.67 ± 81.714	117.98 ± 60.921	3.094	0.002
HDL-C (mg/dL)	52.09 ± 12.662	53.30 ± 12.501	−0.887	0.381
Fasting glucose (mg/dL)	106.97 ± 25.180	104.66 ± 23.798	0.870	0.979
HbA1c (%)	5.90 ± 0.889	5.90 ± 0.859	0.060	0.952
Cardiometabolic risk factors and comorbidities				
Hypertension (Yes)	67 (60.9%)	186 (56.2%)	0.752	0.386
Diabetes mellitus (Yes)	22 (20.0%)	70 (21.1%)	0.074	0.797
Hyperlipidemia (Yes)	38 (34.5%)	145 (43.8%)	2.927	0.088
Kidney disease (Yes)	2 (1.8%)	15 (4.5%)	1.642	0.200
Asthma (Yes)	6 (5.5%)	17 (5.1%)	0.026	0.896
CMRC (≥3 risk factors)	52 (47.3%)	113 (34.1%)	6.082	0.014

Note. Values are presented as *n* (%) for categorical variables and mean ± standard deviation (SD) for continuous variables. Group comparisons were conducted using independent t-tests for continuous variables and chi-square tests for categorical variables. Vitamin D status was categorized as <20 ng/mL (deficient) and ≥20 ng/mL (sufficient). Physical inactivity = not meeting WHO guideline; CMRC = cardiometabolic risk clustering; BMI = body mass index; HDL-C = high-density lipoprotein cholesterol. Statistical significance was set at *p* < 0.05.

**Table 3:** Multivariable linear regression predicting CMRC (*n* = 441).

**Variable**	**B**	**SE**	**β**	**t**	***p***
(Constant)	0.320	0.072	–	4.420	<0.001
Female	−0.037	0.049	−0.038	−0.757	0.449
Age ≥ 75	0.014	0.046	0.014	0.309	0.757
High education	−0.086	0.057	−0.073	−1.502	0.134
Mid-high income	−0.020	0.047	−0.021	−0.439	0.661
Physical activity	−0.029	0.051	−0.025	−0.582	0.561
Vitamin D ≥ 20 ng/mL	−0.121	0.049	−0.108	−2.475	0.014
Current smoker	−0.015	0.070	−0.010	−0.217	0.828
Alcohol use	0.085	0.051	0.083	1.674	0.095
Obesity (BMI ≥ 25)	0.435	0.043	0.437	10.173	<0.001

Note. B = unstandardized regression coefficient; SE = standard error; β = standardized regression coefficient. CMRC = cardiometabolic risk clustering. All predictors were binary-coded as follows: female = 1 (male = 0); age ≥ 75 years = 1 (<75 = 0); high education = 1 (high school graduate or above = 0); mid-to-high income = 1 (middle or higher = 0); physical activity = 1 (meeting WHO guideline), 0 (not meeting); vitamin D ≥ 20 ng/mL = 1 (<20 ng/mL = 0); current smoker = 1 (yes), 0 (no); alcohol use = 1 (yes), 0 (no); obesity = 1 (BMI ≥ 25 kg/m^2^), 0 (BMI <25 kg/m^2^). Model fit: F(9, 431) = 13.29, *p* < 0.001; R^2^ = 0.217 (adjusted R^2^ = 0.201); Durbin–Watson = 2.112. Statistical significance was set at *p* < 0.05.

**Table 4:** Logistic regression with a vitamin D × physical activity interaction and conditional effects of physical activity on CMRC (*n* = 441).

A. Adjusted Logistic Regression with Interaction Term.				
Variable	OR	Z	*p*	95% CI
Constant	0.82	−0.66	0.511	0.46–1.47
Physical activity (X)	8.31	2.38	0.017	1.45–47.61
Vitamin D level (W)	0.99	−0.89	0.375	0.97–1.01
X × W interaction	0.91	−2.83	0.005	0.85–0.97
B. Conditional Effects of Physical Activity at Representative Vitamin D Levels				
Vitamin D level (ng/mL)	OR for PA (X → Y)	Z	*p*	95% CI
17.08 (16th percentile)	1.64	1.31	0.192	0.78–3.47
27.48 (median)	0.61	–1.84	0.066	0.36–1.03
36.98 (84th percentile)	0.25	–3.00	0.003	0.10–0.62

Note. CMRC = cardiometabolic risk clustering; OR = odds ratio; CI = confidence interval. The outcome was binary CMRC (≥3 cardiometabolic risk factors vs. <3). ORs and 95% CIs were obtained by exponentiating logistic regression coefficients. Physical activity (X) was coded as 1 = meeting WHO aerobic physical activity guidelines and 0 = not meeting. Vitamin D (W) refers to serum 25-hydroxyvitamin D (ng/mL; continuous) and was mean-centered prior to creating the interaction term. Accordingly, the main effect of physical activity (X) represents the association between physical activity and CMRC when vitamin D is at its sample mean (i.e., W = 0 after centering). The interaction term (X × W) tests whether the association between physical activity and CMRC varies across levels of vitamin D; an interaction OR < 1 indicates that the association becomes more protective as vitamin D increases (per 1 ng/mL). Conditional effects of physical activity were estimated at representative percentiles of vitamin D (16th, 50th, and 84th). Models were adjusted for sex, age (≥75 vs. <75 years), education, income, smoking, alcohol use, and obesity (BMI ≥25 kg/m^2^). Statistical significance was set at *p* < 0.05.

## Data Availability

Publicly available datasets were analyzed in this study. These data can be found here: https://knhanes.kdca.go.kr (accessed on 11 December 2025).

## Abbreviations

The following abbreviations are used in this manuscript:

25(OH)D	25-hydroxyvitamin D
BMI	Body mass index
CI	Confidence interval
CMRC	Cardiometabolic risk clustering
HDL-C	High-density lipoprotein cholesterol
IRB	Institutional Review Board
KDCA	Korea Disease Control and Prevention Agency
KNHANES	Korea National Health and Nutrition Examination Survey
OR	Odds ratio
WHO	World Health Organization
